# Multimodal imaging in Ig G4- related aortitis: case report

**DOI:** 10.47487/apcyccv.v5i1.317

**Published:** 2024-03-19

**Authors:** Lindsay Benites-Yshpilco, Lucia Barriales-Revilla, Roberto Baltodano-Arellano, Luis Falcón-Quispe, Kelly Cupe-Chacalcaje, Ángela Cachicatari-Beltrán, Gerald Lévano-Pachas

**Affiliations:** 1 Hospital Guillermo Almenara Irigoyen - EsSalud, Lima, Perú. Hospital Guillermo Almenara Irigoyen - EsSalud Lima Perú; 2 Facultad de Medicina, Universidad Nacional Mayor de San Marcos, Lima, Perú Universidad Nacional Mayor de San Marcos Facultad de Medicina Universidad Nacional Mayor de San Marcos Lima Peru; 3 Cardiac imaging area of Cardiology Service, Hospital Guillermo Almenara Irigoyen - EsSalud, Lima, Perú. Cardiac imaging area of Cardiology Service Hospital Guillermo Almenara Irigoyen - EsSalud Lima Perú

**Keywords:** Aortitis, Echocardiography, Doppler, Echocardiography, Transesophageal, Multimodal Imaging, Aortitis, Ecocardiografía Doppler, Ecocardiografía Transesofágica, Imagen Multimodal

## Abstract

We present the case of a 56-year-old patient with fever of unknown origin associated with chest and lumbar pain. Multimodality imaging revealed diffuse peri-aortitis in the thoracic aorta without involvement of the aortic valve, contributing substantially to the diagnosis of Ig G4-associated aortitis. Immunosuppressive therapy was started. Follow-up at five months with cardiac magnetic resonance imaging showed a reduction in the inflammatory process in the thoracic aorta.

## RESUMEN

Presentamos el caso de un paciente de 56 años, con cuadro febril de origen desconocido asociado a dolor torácico y lumbar. La imagen multimodal demostró periaortitis difusa en la aorta torácica sin afectación de la válvula aórtica, lo que contribuyó sustancialmente al diagnóstico de aortitis asociada a Ig4. Se inició tratamiento inmunosupresor. El seguimiento a los 5 meses, la resonancia magnética cardíaca mostró una reducción del proceso inflamatorio en la aorta torácica.

## Introduction

Aortitis is an infrequent cause of chest pain, whose etiology is associated with rheumatologic, infectious or idiopathic diseases. Histopathological vascular exam is the gold standard for diagnosis ^1^^,^^2^. A late diagnosis can be catastrophic, leading to arterial stenosis and multiple organ failure; therefore, starting early therapy should be the principal objective. Currently, non-invasive diagnosis can be done using imaging studies, ensuring an early diagnosis, and avoiding percutaneous invasive procedures ^3^^,^^4^. We present a case report to show the importance of multimodal imaging for the diagnosis of this entity.

## Case Report

A 56-year-old woman came to the emergency room with fever of unknown origin associated with chest and lumbar pain for the last four months. In addition, she had intermittent nausea and vomiting. Her medical history included arterial hypertension, obesity, and deep vein thrombosis of the lower limbs. On physical examination, heart rate was 104 beats per minute, blood pressure was 160/84 mmHg in the right arm and 164/86 mmHg in the left arm. Thoracic auscultation showed no significant findings, and the evaluation of radial, humeral, femoral, and pedal pulses showed no abnormalities.

In laboratory tests, the complete blood count showed no alterations; however, the glomerular sedimentation rate was 53 IU/L, and the C-reactive protein was 22.6 IU/L. On the other hand, the metabolic profile, assessment of syphilis (VDRL), and immunological profile including tests for lupus, ANCA, and IgG4, showed no abnormalities.

**Figure 1:**
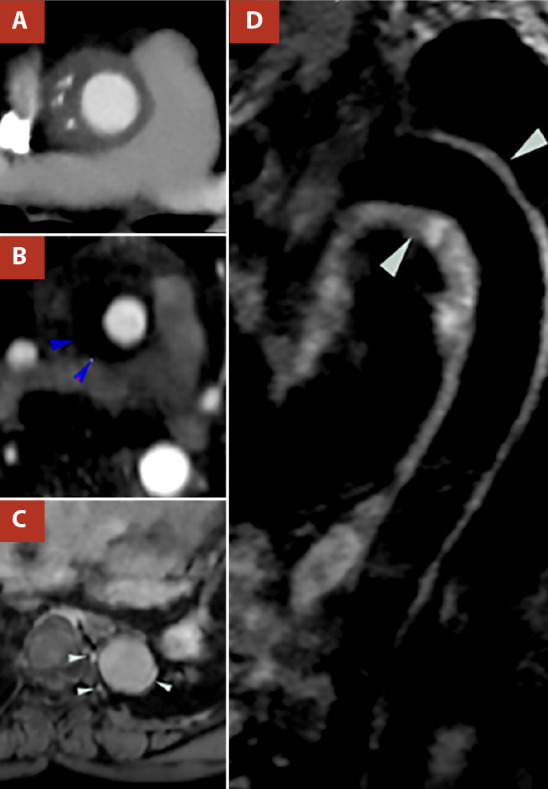
(A)Pre-treatment contrast Computed tomography angiography reformat showed concentric lesion hypoattenuated with 77 HU and thickening of 1.2 cm at the proximal ascending thoracic aorta. (B) Pre-treatment post-contrast resonance angiography showed a thickened ascending aorta wall (13 mm) with a hypointense signal. (blue arrow). (C) Pre-treatment T1w sequence MR in the same patient demonstrated enhancement of the descending aortic wall after administration of intravenous contrast (white arrow). (D) Pre-treatment Double inversion recovery fat sat T2w sequence MR in the same patient at different levels shows a diffuse hyperintense lesion of the aortic wall compatible with periaortic edema (white arrow).

Initially, computed tomography was indicated to explore the thoracic vessels. This study showed a concentric hypo attenuated and thicked lesion of 1.2 cm at the proximal ascending thoracic aorta and dense tissue with scant enhancement surrounding the aorta compatible with peri aortitis extending from the ascending aorta, aortic arch, subclavian, and infrarenal aorta to the common iliac arteries (Figure 1A).

**Figure 2:**
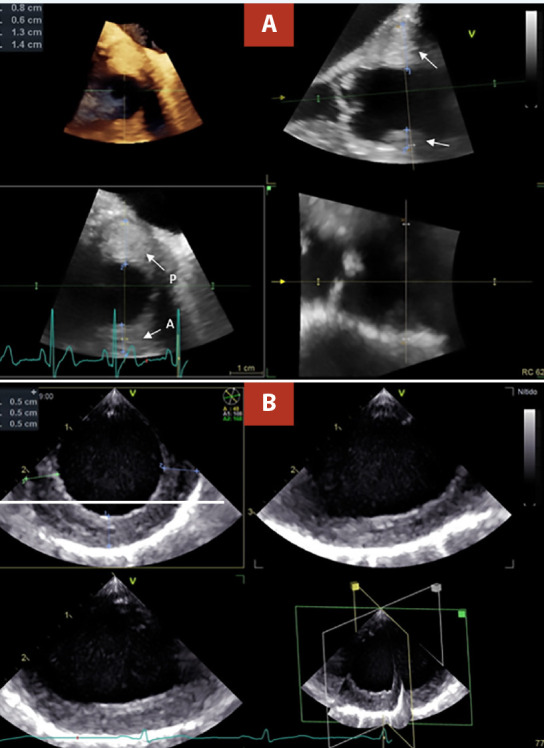
(A) TEE. Triplanar image of the ascending aorta, the arterial wall with asymmetric thickening of 14 mm in posterior aspect “P”, and 8 mm in anterior aspecto “A”. (B) TEE. Triplanar image of the descending thoracic aorta, visualizing arterial wall with homogeneous circumferential thickening of 5mm.

With those findings, transesophageal echocardiography was recommended, which clearly showed diffuse wall thickening from the proximal ascending aorta to the proximal third of the descending aorta (maximum thickness of 15 mm). The circumferential involvement was evidenced in the tri-planar image of the thoracic aorta. (Figure 2, video 1). In the aortic valve exploration, mild aortic regurgitation was found, and in the rest of the study, no relevant alterations were reported **(**video 2**)**.

Finally, cardiac magnetic resonance imaging (MRI), in the T1 sequence showed a thickened ascending aorta wall (13mm) with a hypointense signal (Figure 1B) and enhancement of the descending aortic wall after administration of intravenous contrast and in the T2 sequence, evidenced a hyperintense area circumferential to the aorta, extending from the ascending aorta, the aortic arch, and the descending aorta, compatible with an inflammatory process (Figure 1D). Unfortunately, a percutaneous biopsy was not performed due to the logistic and expertise limitations of the center.

**Figure 3:**
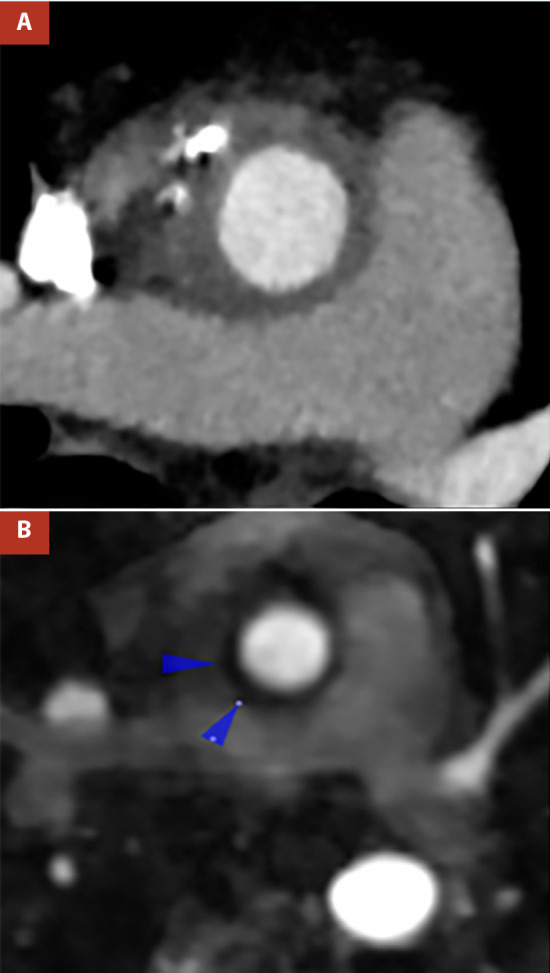
(A) Contrast Computed tomography angiography after corticosteroid therapy revealed decreased thickening of the aortic wall of 8.5 cm and attenuation of 50UH. (B) Post-treatment post-contrast resonance angiography revealed a decrease in aortic wall thickness (8mm) with hypointense signal. (blue arrow)

Once the findings were determined, the rheumatology department, with clinical and mainly radiological criteria, made the diagnosis of aortitis associated with Ig G4; for this reason, the patient began to receive prednisone 20 mg twice a day, and one month later azathioprine daily. At the fifth month of treatment, she reported no fever and no chest or lumbar pain. On the other hand, CT angiography and cardiac MRI revealed an evident decrease in aortic thickness (Figure 3).

## Discussion

Inflammation of the aortic wall layers can be caused by a wide variety of systemic diseases, from rheumatologic disorders, and infectious diseases to idiopathic entities ^1^^,^^2^. Cardiovascular imaging plays an important role in the diagnosis and identification of complications ^3^.

Computed tomography is a non-invasive procedure, which explores the whole thoracic aorta and its different morpho-structural presentations such as inflammatory vasculitis, aneurysmal change, and pseudotumor formation. It is the choice test in the detection of aortitis, due to its high sensitivity and specificity (95% and 100% respectively), and it can also be synchronized with electrocardiography, particularly for evaluation of coronary arteries, avoiding percutaneous invasive procedures ^4^.

MRI can detect complications of the aortic wall such as thickening, ulceration, and formation of pseudoaneurysms and aneurysms, with greater sensitivity than tomography. Likewise, it is useful in the differentiation of disease stages, the T1 sequence shows hypointense images related to chronic periarteritis, on the other hand, the T2 sequence shows hyperintense images in the early and active stages of the disease due to inflammatory edema, while the low-intensity signal is commonly observed in the late fibrotic stages ^5^.

Transthoracic echocardiography (TTE) in this entity, is particularly relevant in determining the presence of aortic regurgitation and providing signs of dilatation or thickening of the ascending aorta ^6^. 

Transesophageal echocardiography (TEE) provides realistic and high temporal-spatial resolution images of the thoracic aorta, except for the distal third of the ascending aorta ^6^. Additionally, it defines with high-precision, pathologies such as atherosclerosis including the presence of plaques, aortic dissection, and inflammatory processes as in our case ^6^. An additional utility of the TEE is to pinpoint the location of the structural abnormality to guide the biopsy and even the surgical act, likewise, TEE can account for immediate complications following the intervention ^7^^,^^8^.

New imaging tools are being developed such as nuclear medicine imaging with fluorine-18 fluorodeoxyglucose (18-FDG), with high sensitivity, especially in the early phase of the disease ^9^, by evaluating the increase of glucose uptake in the vessel walls, estimated with a variable sensitivity between 56 to 100%.

Aortitis is part of a long list of differential diagnoses of chest pain ^1^, as was the case in our patient. IgG4-related aortitis can be difficult to diagnose in the absence of other organ involvement ^2^. As mentioned above, histological examinations were not performed, so the rheumatologic medical team determined to initiate treatment for this entity based primarily on clinical and radiological findings.

Among the differential diagnoses is giant cell arteritis; however, the patient did not present headache or pain in the temporal artery, and the diagnostic imaging evidenced an absence of involvement of the extracranial carotid branch ^1^. Takayasu’s disease was excluded by physical examination; there was no difference in pulse in both arms, and the tomographic findings were diffuse and not obstructive. On the other hand, systemic lupus was not considered because of negative clinical and serological markers ^2^. Syphilitic aortitis was eliminated from the list of diagnoses due to the absence of epidemiological history, negative VDRL test, and non-localized aortic involvement ^10^. Another infectious aortitis was ruled out because the patient did not present a septic aspect, a hemogram without relevant findings, and the disease duration was prolonged ^11^.

In conclusion, IgG4 aortitis is a systemic inflammatory disease that can cause from non-specific symptoms to the involvement of large vessels, which can potentially generate tragic complications such as myocardial ischemia, aortic dissection, or rupture.

Multimodal imaging has an essential role in determining the diagnosis of IgG4-associated aortitis, even more so when a histological study is not available. Its usefulness extends to follow-up, to determine response to immunosuppressive and anti-inflammatory therapy.
